# A Ground-Based Visual System for UAV Detection and Altitude Measurement Deployment and Evaluation of Ghost-YOLOv11n on Edge Devices

**DOI:** 10.3390/s26010205

**Published:** 2025-12-28

**Authors:** Hongyu Wang, Yifeng Qu, Zheng Dang, Duosheng Wu, Mingzhu Cui, Hanqi Shi, Jintao Zhao

**Affiliations:** School of Automation and Intelligent Sensing, Shanghai Jiao Tong University, Shanghai 200240, China

**Keywords:** UAV detection, edge AI, YOLOv11, monocular ranging, Kalman filter, embedded deployment

## Abstract

**Highlights:**

**What are the main findings?**

- We propose Ghost-YOLOv11n, a lightweight UAV detector that reduces computational cost by 12.7% while achieving 98.8% mAP_0.5_ on a comprehensive dataset of 8795 images.
- The system, deployed on a low-power LuBanCat4 edge device with NPU acceleration, achieves 20 FPS and maintains altitude measurement errors within 10% up to 30 m using a monocular vision- and EKF-based approach.

**What are the implications of the main findings?**

- This work provides a cost-effective, edge-deployable solution for ground-based UAV surveillance, enabling long-term, low-power operation suitable for protecting critical infrastructure.
- It establishes a practical benchmark for integrated detection and altitude measurement systems in real-world ground-to-air scenarios, bridging the gap between algorithm design and system-level deployment.

**Abstract:**

The growing threat of unauthorized drones to ground-based critical infrastructure necessitates efficient ground-to-air surveillance systems. This paper proposes a lightweight framework for UAV detection and altitude measurement from a fixed ground perspective. We introduce Ghost-YOLOv11n, an optimized detector that integrates GhostConv modules into YOLOv11n, reducing computational complexity by 12.7% while achieving 98.8% mAP_0.5_ on a comprehensive dataset of 8795 images. Deployed on a LuBanCat4 edge device with Rockchip RK3588S NPU acceleration, the model achieves 20 FPS. For stable altitude estimation, we employ an Extended Kalman Filter to refine measurements from a monocular ranging method based on similar-triangle geometry. Experimental results under ground monitoring scenarios show height measurement errors remain within 10% up to 30 m. This work provides a cost-effective, edge-deployable solution specifically for ground-based anti-drone applications.

## 1. Introduction

The strategic impact of unmanned aerial vehicles (UAVs) in modern conflicts underscores their potential as asymmetric threats to civilian infrastructure [1,2]. The protection of critical ground installations from unauthorized drone incursions has therefore emerged as a pressing security challenge worldwide. Airports, power stations, and government facilities represent static assets vulnerable to low-altitude, small-scale UAVs that can evade conventional radar detection. This reality necessitates the development of cost-effective, autonomous ground-based perception systems capable of reliable detection and localization of aerial threats. Unlike mobile airborne platforms, fixed ground surveillance presents a distinct set of operational constraints and requirements, where long-term, low-power operation and accurate altitude estimation are paramount for effective threat assessment and response.

It is important to position this work in relation to our recent research on ADG-YOLO [3]. That study was designed for airborne, drone-to-drone detection, optimizing for high performance in dynamic environments from a mobile platform. In contrast, the present work focuses squarely on the ground-to-air monitoring scenario. This context imposes fundamentally different requirements, prioritizing power efficiency, long-term operational stability, and robustness to complex static backgrounds over raw performance. Thus, the contributions of this paper are complementary, exploring a dedicated technical pathway optimized for static ground defense nodes.

The principal challenges for a ground-deployed system are threefold: (1) Although lightweight models are validated on embedded GPUs, their performance and stability on ultra-low-power terminals that rely heavily on NPU acceleration remain underexplored; (2) The accuracy of geometric ranging is highly susceptible to detection jitter and false positives in single-frame inference, adversely affecting continuous measurement; (3) There is a scarcity of empirical studies on lightweight, end-to-end solutions that achieve robust integration of detection and height measurement on resource-constrained ground terminals, and are rigorously tested in real-world ground-side scenarios.

To address these challenges, this paper proposes and implements Ghost-YOLOv11n, a unified framework for real-time UAV detection and altitude measurement specifically designed for fixed ground stations. The main contributions of this work are distinct in their focus on ground-side deployment:

1. We construct a comprehensive UAV detection dataset of 8795 images, with an emphasis on perspectives and scenarios relevant to ground observation;
2. We propose the Ghost-YOLOv11n model, which achieves a significant reduction in computational cost through a unified GhostConv design, prioritizing efficiency for computation-limited ground nodes;
3. We demonstrate efficient edge deployment and acceleration on a low-power LuBanCat 4 board, validating the system’s operation in a practical ground-side form factor;
4. We implement a real-time monocular altitude estimation pipeline, stabilized by an Extended Kalman Filter, specifically tailored for the ground-to-air measurement context;
5. We construct and empirically evaluate a complete end-to-end system, testing and validating its performance in outdoor, ground-based environments to establish a performance benchmark for this application.

The outcomes of this study offer a feasible and practical technical pathway for deploying efficient, low-cost ground-side UAV monitoring and countermeasure systems on resource-constrained edge devices.

Structure of the paper: The remainder of this paper is organized as follows. Section 3 details the proposed methodology, including the dataset construction, the Ghost-YOLOv11n architecture, the edge deployment pipeline, camera calibration and the monocular ranging method. Section 4 presents the experimental setup, results, and analysis of detection performance, altitude measurement accuracy, and power consumption. Section 5 provides a discussion of the results, including comparative analysis and error investigation. Finally, Section 6 concludes the paper and suggests directions for future work.

## 2. Related Works

### 2.1. UAV Sensing and Detection Modalities

Reliable detection is the first step in counter-UAV systems. Traditional active sensors like radar [4] and LiDAR [5] offer high accuracy but are often constrained by high cost, power consumption, and size for distributed deployment. Ultrasonic sensors [6] have limited range. Alternative passive modalities have been explored to address these limitations. Radio frequency scanning systems can detect and classify drones by their communication signals [7], while acoustic-based systems utilize the distinct sound signatures of UAV propellers [8]. These provide complementary, low-cost options but can be affected by environmental noise and signal interference. In contrast, vision-based sensing [9] provides rich contextual information, is inherently passive, and benefits from continuously falling hardware costs, making it a highly promising modality for pervasive, intelligent surveillance networks.

### 2.2. Monocular Vision-Based Ranging

Among vision-based methods, monocular vision is particularly attractive for embedded systems due to its minimal hardware setup [10,11]. The core challenge is recovering metric scale from a single view, leading to two distinct research paradigms.

Relative Depth Estimation: This data-driven paradigm employs deep learning to predict dense, pixel-wise depth maps, excelling at scene understanding. The field has evolved rapidly from earlier convolutional architectures [12,13,14,15] to contemporary methods incorporating Vision Transformers for global context, such as the state-of-the-art RMTDepth framework for UAV videos [16]. A critical limitation of this paradigm is that it typically outputs up-to-scale depth, lacking the absolute physical scale required for direct distance measurement in security applications.

Absolute Distance Estimation: This category aims to obtain measurable distances, primarily through two approaches. Learning-based regression methods directly map image features to distance but require extensive training and generalize poorly [17]. Geometry-based methods, such as the similar triangle model [18,19,20], leverage projective geometry and prior knowledge of the target’s physical dimensions. They are computationally efficient and interpretable but are highly dependent on the accuracy and stability of the target bounding box provided by a detector. While foundational geometric techniques like Inverse Perspective Mapping (IPM) [21,22] were influential, the similar triangle model remains predominant for its simplicity and effectiveness in constrained systems.

### 2.3. Object Detection for UAVs

Accurate and efficient object detection is the critical prerequisite for vision-based UAV monitoring. The field has been shaped by the evolution from two-stage detectors like the R-CNN series [23,24,25] to the one-stage YOLO family [26,27,28,29,30,31,32,33,34], which offers a superior speed–accuracy trade-off for real-time applications. For the specific challenge of detecting small UAVs, significant optimizations have been made to recent YOLO architectures through lightweight convolutions, attention mechanisms, and enhanced feature fusion networks [35,36,37,38,39,40]. Specialized models like SEB-YOLOv8s demonstrate focused improvements for real-time unauthorized UAV detection [41]. However, a notable gap exists: many studies primarily report performance on desktop GPUs or high-power embedded modules, while systematic optimization and evaluation for ultra-low-power NPU accelerators, which are crucial for long-duration field deployment, remain less explored.

### 2.4. Identified Research Gap and Our Position

In summary, while substantial progress exists in individual components—alternative sensing modalities, depth estimation networks, and efficient detectors—a significant gap persists at the system integration level for ground-based applications. First, there is a disconnect between advanced detection algorithms and their practical deployment on power- and compute-limited edge hardware suitable for fixed sites. Second, many studies treat detection and ranging as separate tasks; a tightly coupled, co-designed lightweight pipeline that ensures detection stability feeds into robust geometric ranging is underexplored. Third, comprehensive evaluation often lacks the ground-based perspective and real-world validation critical for this scenario, unlike works focusing on drones as sensing platforms [42].

This work aims to bridge these gaps by presenting Ghost-YOLOv11n, an end-to-end, ground-optimized framework. Our contributions directly address the identified limitations: we co-design a detector for NPU-edge efficiency, integrate it with a stabilized monocular ranging pipeline, and provide extensive empirical validation in realistic ground-side scenarios.

## 3. Materials and Methods

In this section, we first constructed a customized aerial image dataset of unmanned aerial vehicles (UAVs) and employed an improved YOLOv11n algorithm in combination with the Extended Kalman Filter (EKF) for real-time detection of target UAVs. The trained model was subsequently deployed on an edge computing device, the LuBancat 4 development board (EmbedFire, Dongguan, China), enabling lightweight inference. Furthermore, we proposed a UAV height estimation method based on the principle of similar triangles, which utilizes the known physical dimensions of the target UAV and the width of the YOLO detection bounding box to estimate the flight height of UAVs with known sizes.

### 3.1. Ghost-YOLOv11n-Based UAV Detection and Edge Deployment

#### 3.1.1. Datasets

**Figure 1:**
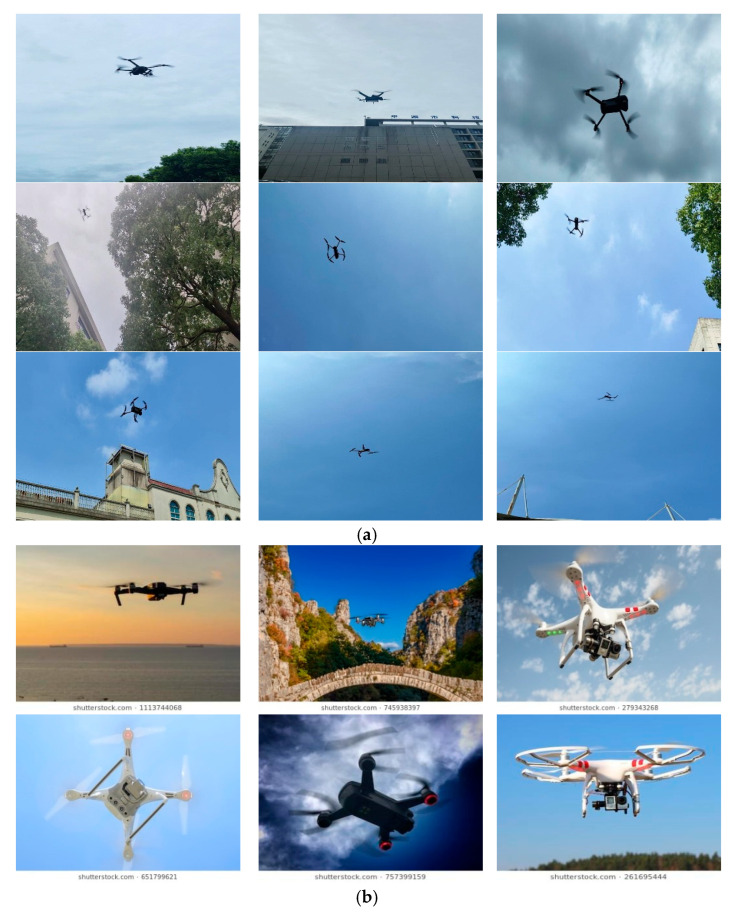
Partial images of dataset. (**a**) Custom-built subset. (**b**) Generalization subset [43].

In this study, we constructed a comprehensive UAV detection dataset comprising a total of 8795 high-resolution images. The dataset systematically integrates a self-collected target subset (6163 images) and an open-source generalization subset (2664 images). Representative samples from both subsets are shown in Figure 1. Among them, 1495 images were randomly selected to form the validation set for model evaluation.

The custom-built subset includes two types of drones, the DJI 3TD and the DJI Mavic 3E, which were flown at various altitudes and environments as targets for image capture. During the capture process, handheld cameras were used to photograph the drones from multiple angles, ensuring that different perspectives were covered, including low-angle, side, and upward views. The camera angles were adjusted according to the flight path and position of the drones to acquire a more diverse set of images. The flight altitudes of the drones spanned low, medium, and high altitudes. When flying at low altitudes, the drones were close to the ground, with the background possibly including ground features or buildings; at higher altitudes, the drones were further from the ground, with the background consisting of expansive sky or distant buildings. These variations in flight altitude and background provide the dataset with rich diversity. The subset of capture scenes encompasses several environments, including open skies, urban buildings, trees, and indoor ceilings, thus presenting complex and variable backgrounds for drone image recognition tasks. To enhance the adaptability of the dataset, the captures were performed under various weather conditions, including light rain, overcast, cloudy, and sunny conditions. Each weather scenario results in different lighting and visual effects on the images. For instance, in light rain and overcast conditions, the images may be affected by low lighting and raindrops, while sunny weather offers optimal shooting conditions with higher image clarity.

The generalization subset was sourced from publicly available UAV image datasets on the CSDN object detection platform [43]. It features quadrotor and hexacopter UAVs from well-known brands like DJI captured in various environments such as urban settings, rural areas, highways, and industrial zones. The dataset also includes images taken under challenging weather conditions like bright sunlight, fog, and rain. All images were annotated using professional tools, following the YOLOv11 format, with normalized center coordinates (*x*,*y*) and the relative width *w* and height *h* for each bounding box.

#### 3.1.2. Basic YOLOv11 Architecture

YOLOv11, as an upgraded version of YOLOv8, maintains the overall three-part architecture comprising the Backbone, Neck, and Head, while achieving multi-task performance optimization through module-level redesign.

First, in the backbone network, YOLOv11 replaces the C2f module used in YOLOv8 with a newly designed C3K2 module. The C3K2 module is an improved version of the traditional CSP Bottleneck structure, employing a dual-branch parallel convolution design: the input feature map is split into two parts—one directly propagates shallow features, while the other passes through a configurable C3k submodule for deep feature extraction. The submodule supports the use of convolutional kernels of different sizes (e.g., 3 × 3, 5 × 5), and the two branches are ultimately fused via channel concatenation. By adjusting hyperparameters such as the repetition count *n*, the number of grouped convolutions *g*, and the channel expansion ratio e, the C3K2 module achieves a dynamic balance between computational efficiency and feature representation capability. Compared to the C2f module, C3K2 offers enhanced receptive field expansion and better adaptability to large-scale targets. Second, in the neck network, a novel C2PSA (Cross Stage Partial with Pyramid Squeeze Attention) module is introduced. This module integrates the CSP structure with a pyramid squeeze attention mechanism, extracting multi-scale features via parallel convolutions of 3 × 3, 5 × 5, and 7 × 7 kernel sizes. Additionally, a squeeze-and-excitation (SE) mechanism is employed for dynamic channel-wise weighting, which not only improves the detection of occluded targets but also effectively reduces computational complexity through the CSP design. The detection head is reconstructed with lightweight principles, incorporating the C3K2 module and depthwise separable convolution to significantly reduce computational cost while maintaining high feature resolution. Furthermore, a C2PSA module is appended to the end of the backbone network (after the SPPF module) to enhance the integration of global contextual information.

#### 3.1.3. Lightweight GhostConv Module

To meet the stringent requirements of long-term, low-power deployment on fixed ground nodes, this study adopts the GhostConv module [44] as the foundational building block, systematically replacing standard convolutions throughout the network. This represents a deliberate architectural shift from the multi-component, performance-maximizing design of ADG-YOLO [3] to a unified, stability-oriented design for ground-based systems. GhostConv, introduced by Huawei Noah’s Ark Lab, is a lightweight convolution module designed to reduce model parameters and computational cost through an efficient feature generation process. More importantly, its homogeneous and streamlined architecture is inherently suited to the stability and efficiency demands of resource-constrained edge devices performing persistent surveillance tasks.

The core principle of GhostConv is described in Equations (1)–(3):

(1) Y=[ϕ(X),ψ(X)]

(2) $$\phi (X)=X*K_{primary}$$

(3) $$\psi (X)=f_{linear}(X)*K_{cheap}$$

**Figure 2:**
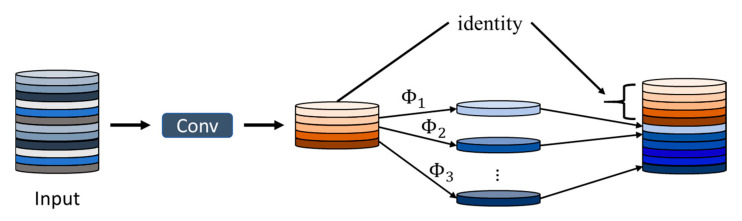
The Ghost module [3].

In these equations, X denotes the input feature map, $K_{\mathrm{primary}}$ and $K_{\mathrm{cheap}}$ represent the primary convolutional kernel and the lightweight convolutional kernel, respectively, and $f_{\mathrm{linear}}(\cdot )$ denotes a depthwise separable linear transformation function. As illustrated in Figure 2, the GhostConv module divides the input feature map into two branches: one branch generates the “primary features” using standard convolution, while the other produces “ghost features” through inexpensive linear operations such as depthwise separable convolutions. The two branches are then concatenated to form the final output. This design effectively exploits feature redundancy, enabling the generation of a comparable number of output feature maps with significantly fewer convolutional kernels. As a result, the number of parameters and GFLOPs is greatly reduced while preserving a representation capacity similar to that of standard convolutions.

Critically for deployment, GhostConv demonstrates excellent structural compatibility with native YOLOv11 modules. It maintains consistent input and output channel dimensions with the original Conv modules, allowing for seamless integration into components such as the C3K2 and C2PSA modules. Furthermore, within the C3K2 module, GhostConv employs a channel-splitting strategy to further compress parameter size. This design coherence results in a more predictable and stable computational graph on the edge NPU, which is a vital characteristic for ensuring reliable, long-term operation of a ground-based system. This enables GhostConv to significantly reduce computational overhead while ensuring a favorable balance between detection accuracy and inference speed under real-world deployment constraints.

#### 3.1.4. Ghost-YOLOv11n Model

**Figure 3:**
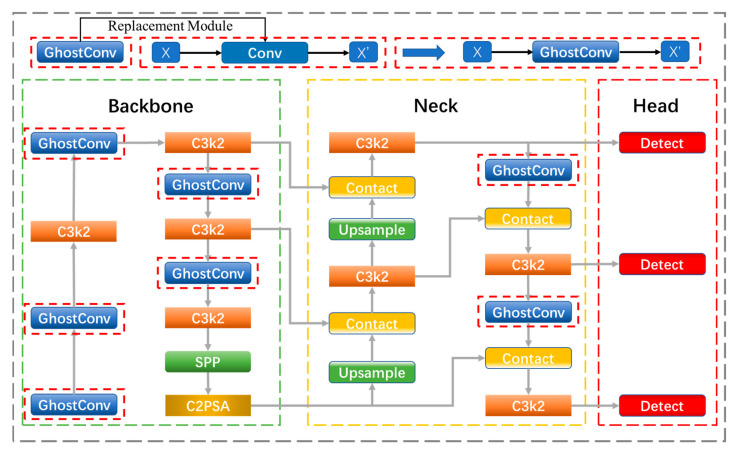
Ghost-YOLOv11 Network Architecture (the red dashed box indicates the replaced Ghost module).

As shown in Figure 3, in the Ghost-YOLOv11n model, the GhostConv module is systematically deployed as the core unit replacing standard convolutions across all critical stages of the network. It is integrated throughout the backbone for down-sampling at key transitions such as the P1/2, P2/4, P3/8, P4/16 and P5/32 stages, within the feature fusion layers for efficient channel adjustment before concatenation operations, and in the detection heads for final feature compression prior to the output layers. This module is built upon the lightweight design philosophy of GhostNet, aiming to reduce redundant feature computations to lower both the parameter count and computational complexity of the model. Specifically, GhostConv first employs a small number of standard convolutions to generate primary feature maps, and then produces approximate “ghost features” through a series of inexpensive linear operations such as depthwise separable convolutions. The two sets of features are subsequently merged to form the complete output. This mechanism not only significantly reduces computational cost while maintaining the model’s representational capability, but also markedly improves deployment efficiency in resource-constrained environments.

Therefore, the comprehensive integration of GhostConv across the backbone, neck and head is one of the key enablers for Ghost-YOLOv11n to achieve lightweight and efficient object detection, making it particularly suitable for real-time applications on mobile or edge computing devices.

#### 3.1.5. Model Conversion and Edge Deployment

The pipeline for model conversion and deployment onto the LubanCat4 edge device mirrors the procedure outlined in [3]. Edge computing devices offer several significant advantages, including low latency, real-time responsiveness, bandwidth savings, and enhanced data privacy and security. By performing computation and inference tasks locally near the data source, these devices effectively reduce latency and network load caused by data transmission, while mitigating privacy risks associated with uploading sensitive information to the cloud. In addition, edge devices are typically compact and energy-efficient, making them well-suited for resource-constrained scenarios or applications with strict energy-efficiency requirements. Their deployment flexibility and cost-effectiveness further enhance their practicality. In application scenarios such as drone-based surveillance, edge computing devices enable real-time, on-device target recognition, characterized by lightweight design, low cost, and ease of integration. These features allow widespread deployment on mobile platforms, border patrol sites, and other complex environments, meeting dual demands for portability and rapid response. Common types of edge computing devices are listed in Table 1.

**Figure 4:**
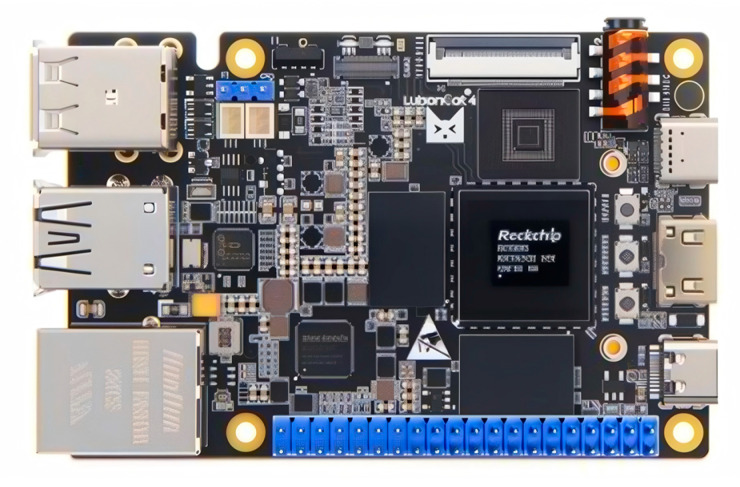
LubanCat4 Development Board.

After a comprehensive evaluation of factors such as size, weight, scalability, and cost, the YOLOv11 model was deployed on the LuBancat4 development board equipped with 4 GB of RAM. This board features the Rockchip RK3588S processor and integrates an AI accelerator NPU with 6 TOPS of computing power, supporting mixed-precision inference with INT4, INT8, and INT16. Additionally, the board is equipped with a mini HDMI port and a USB camera interface, and has a total weight of approximately 62 g, as shown in Figure 4. Due to the limited CPU computing power, efficient inference requires converting the original .pt model into the .rknn format in order to fully leverage the acceleration capabilities of the onboard NPU. As shown in Table 1, these are common edge computing devices.

**Figure 5:**
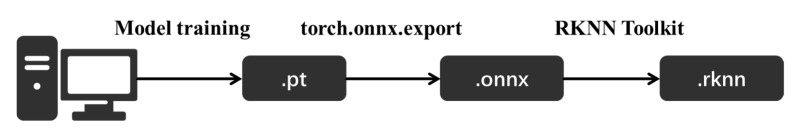
YOLOv11n Model Conversion Process Diagram [3].

The YOLOv11 model trained on the PC is initially saved in the .pt format, which must be converted to the .rknn format before deployment. The conversion process involves first exporting the model to the .onnx format using PyTorch’s torch.onnx.export function. Subsequently, the RKNN Toolkit (Rockchip, Fuzhou, China) is used to convert the .onnx model into the .rknn format. The overall conversion pipeline is illustrated in Figure 5. Once the conversion is complete, the model can be deployed to the development board for real-time UAV target detection in image streams. Benefiting from the hardware acceleration capability of the NPU, this process enables efficient image processing and accurate UAV detection, thereby ensuring real-time recognition and rapid response in practical application scenarios.

It is worth noting that the NPU integrated in the Lubancat 4 development board does not provide hardware acceleration for the Sigmoid or Silu activation functions. As a result, computations involving these functions must be executed by the CPU, potentially leading to inference latency bottlenecks. To address this issue, the Silu function is typically replaced with the more computationally efficient ReLU function during the model training phase, enabling the entire model to be executed by the NPU during deployment and thereby improving inference speed. However, the use of the ReLU function may lead to a reduction in detection accuracy. Therefore, it is meaningful to compare different activation functions in terms of both accuracy and inference speed. A detailed experimental analysis of this comparison is provided in Section 4.1.

#### 3.1.6. Target Monitoring Based on YOLOv11 Detection and EKF Tracking

The EKF tracking module employed in this study, including the state vector definition and the filtering process, follows the same formulation as detailed in our prior work [3]. In [3], this EKF framework was established to achieve robust tracking for airborne platforms. The YOLOv11 model is employed to extract the bounding box of the target from each video frame in real time, including the center coordinates and size information. To enable temporal filtering and trajectory prediction of the detected target, we model the target’s state vector as a six-dimensional vector $x={\left[c_{x},c_{y},v_{x},v_{y},w,h\right]}^{T}$, where $\left(c_{x},c_{y}\right)$ denotes the center coordinates of the bounding box, $\left(v_{x},v_{y}\right)$ represents the horizontal and vertical velocities of the center point, and *w* and *h* correspond to the width and height of the bounding box, respectively.

The state transition model assumes constant velocity motion and that the bounding box size remains unchanged over short time intervals. The corresponding state transition matrix is defined as follows:

(4) $$F=\left[\begin{array}{cccccc} 1 & 0 & \Delta t & 0 & 0 & 0\\ 0 & 1 & 0 & \Delta t & 0 & 0\\ 0 & 0 & 1 & 0 & 0 & 0\\ 0 & 0 & 0 & 1 & 0 & 0\\ 0 & 0 & 0 & 0 & 1 & 0\\ 0 & 0 & 0 & 0 & 0 & 1 \end{array}\right]$$

The observation output of YOLOv11 consists of the target’s bounding box information in the image, denoted as ${\left[c_{x},c_{y},w,h\right]}^{T}$. The mapping relationship between this observation and the state vector is defined by the observation matrix as follows:

(5) $$H=\left[\begin{array}{cccccc} 1 & 0 & 0 & 0 & 0 & 0\\ 0 & 1 & 0 & 0 & 0 & 0\\ 0 & 0 & 0 & 0 & 1 & 0\\ 0 & 0 & 0 & 0 & 0 & 1 \end{array}\right]$$

The execution of the EKF consists of two stages: prediction and update. First, during the prediction stage, the target’s state and covariance are predicted based on the current state and the state transition model:

(6) $$\left\{\begin{array}{l} x^{\prime }=F\cdot x,\\ P^{\prime }=F\cdot P\cdot F^{T}+Q \end{array}\right.$$

Here, *Q* represents the process noise covariance matrix.

After obtaining the new observation *z* provided by YOLOv11, the update step is performed:

Residual calculation:

(7) $$y=z-H\cdot x^{\prime }$$

Kalman gain calculation:

(8) $$K=P^{\prime }\cdot H^{T}\cdot {\left(H\cdot P^{\prime }\cdot H^{T}+R\right)}^{-1}$$

State update:

(9) $$x=x^{\prime }+K\cdot y$$

Covariance update:

(10) $$P=(I-K\cdot H)\cdot P^{\prime }$$

Here, *P* is the observation noise covariance matrix, and *I* is the identity matrix.

The integration of the Extended Kalman Filter module effectively mitigates position jitter and sporadic false detections inherent in single-frame YOLOv11 detections, thereby yielding smoother target position estimates and enhancing tracking stability and robustness over consecutive frames.

### 3.2. Monocular Ranging for UAVs Using Similar Triangles

#### 3.2.1. Distance Measurement Method Based on Similar Triangles

The monocular ranging principle based on similar triangles, along with its geometric derivation and associated figures, is consistent with that presented in [3]. In [3], this geometric model provided a computationally efficient means for relative distance estimation between drones. Similar triangles represent a fundamental concept in geometry, characterized by the property that corresponding sides are proportional when corresponding angles are equal. Based on this geometric principle, if the real-world dimensions of an object are known and its projected size in the image can be measured, the distance between the object and the camera can be inferred using the similarity relationship of triangles.

**Figure 6:**
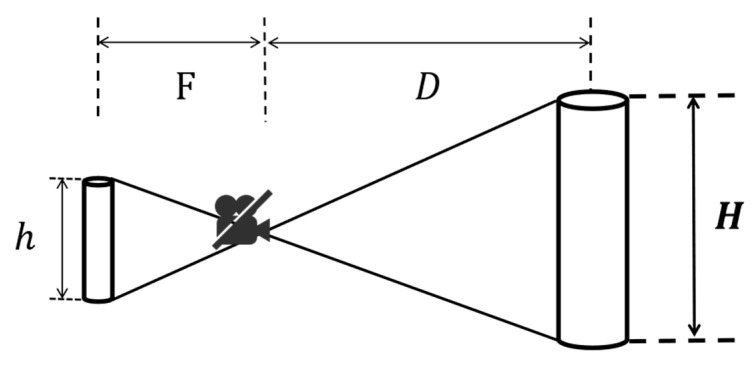
Similar-Triangle Distance Measurement Diagram.

As illustrated in Figure 6, let the actual size of the target object be denoted as *H*, the camera focal length as *F*, the projected size on the image plane as *h*, and the perpendicular distance between the object and the camera as *D*. According to the principle of similar triangles, the following relationship holds:

(11) $$\frac{F}{D}=\frac{h}{H}$$

Consequently, the distance to the target can be calculated using the following formula:

(12) $$D=\frac{H\cdot F}{h}$$

#### 3.2.2. Monocular Vision-Based Drone Height Measurement Principle

In this study, a monocular vision-based method is proposed for estimating the flight height of a target UAV by combining prior knowledge of its physical dimensions with the bounding box output of the YOLOv11 detection algorithm. When a UAV target is detected, YOLOv11 generates a bounding box, whose width is typically used as the image-projected width of the UAV. As a result, the pixel width *w_p_* of the UAV in the image can be obtained and subsequently converted into its projected width *w* on the image plane.

**Figure 7:**
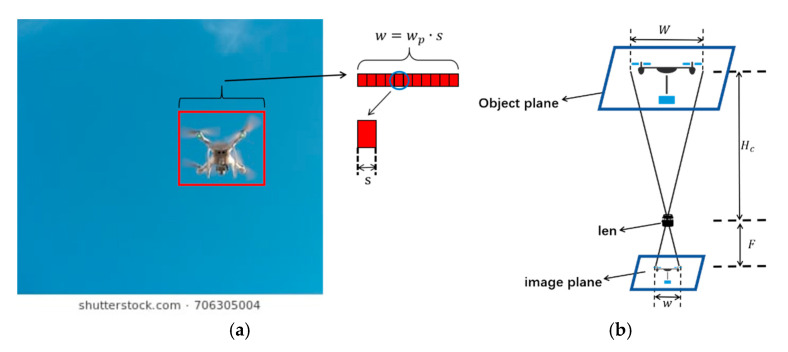
(**a**) UAV Projection Width *w* Diagram. (**b**) UAV Height Measurement Principle Diagram.

As illustrated in Figure 7a, the width of the bounding box *w* is obtained by multiplying the number of pixels *w_p_* by the physical size of a single pixel *s* of the camera sensor, that is,

(13) $$w=w_{p}\cdot s$$

The UAV flight height estimation method is illustrated in Figure 7b. Given the camera’s focal length *F* and the known width *W* of the target drone, the vertical distance $H_{c}$ between the target and the camera (i.e., the flight height) can be calculated using the principle of similar triangles as follows:

(14) $$H_{c}=\frac{W\cdot F}{w}$$

The camera focal length *F* can be obtained using the camera calibration tool described in Section 3.3 of this study.

### 3.3. Camera Calibration Method and Parameter Acquisition

To achieve monocular vision-based drone flight altitude measurement, it is necessary to first obtain the camera’s intrinsic matrix and distortion parameters. In this study, the classical Zhang’s Calibration Method was employed, utilizing MATLAB’s (MATLAB R2023a) Camera Calibration Toolbox for offline camera calibration.

#### 3.3.1. The Principle of Calibration

Zhang’s Calibration Method is a camera parameter estimation approach based on multiple planar images [45]. The basic principle involves capturing two-dimensional checkerboard patterns with known structures and extracting the corner coordinates from the images. These coordinates are then mapped to the actual physical coordinates to solve for the camera’s intrinsic parameters, extrinsic parameters, and distortion coefficients. The camera’s intrinsic matrix *K* is represented as follows:

(15) $$K=\left[\begin{array}{ccc} f_{x} & 0 & c_{x}\\ 0 & f_{y} & c_{y}\\ 0 & 0 & 1 \end{array}\right]$$

where $f_{x}$ and $f_{y}$ represent the pixel focal lengths in the horizontal and vertical directions (in pixels), and $c_{x}$ and $c_{y}$ are the coordinates of the principal point.

Additionally, lens distortion primarily includes radial distortion and tangential distortion. This study considers only the first two orders of radial distortion, with parameters *k*_1_ and *k*_2_.

#### 3.3.2. Calibration Process

**Figure 8:**
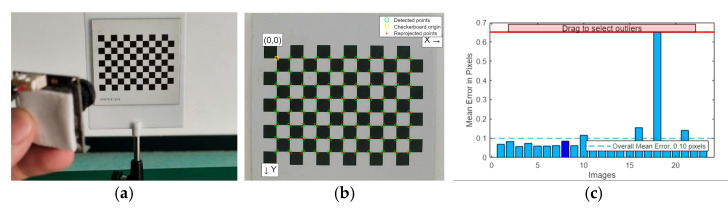
Camera Calibration Process Flow. (**a**) Image Acquisition. (**b**) Corner Detection. (**c**) Error Analysis.

The calibration process was performed using MATLAB’s Camera Calibration Toolbox as follows: During the image acquisition phase, the camera captured 23 images from different angles, each containing a checkerboard pattern with a square size of 5 mm. In the corner extraction phase, the internal corner coordinates of each image were automatically detected and refined to subpixel accuracy. Parameter estimation was carried out using MATLAB’s estimateCameraParameters function, which yielded the intrinsic matrix, distortion coefficients, and reprojection errors. The error analysis results showed that the average reprojection error was approximately 0.1 pixels, indicating high calibration accuracy. Figure 8 illustrates the processes of image acquisition, corner extraction, and error analysis during the camera calibration.

#### 3.3.3. Calibration Results

The final obtained camera intrinsic matrix is:

(16) $$K=\left[\begin{array}{ccc} 3993.12 & 0 & 312.36\\ 0 & 3996.46 & 247.98\\ 0 & 0 & 1 \end{array}\right]$$

The radial distortion parameters are: $k_{1}=-0.6044,\;k_{2}=2.3337$.

The focal length obtained from Equation (16) represents the pixel focal length. To obtain the actual focal length of the camera, it must be multiplied by the physical size of a single pixel *s*. This parameter set is used for image undistortion processing and provides the focal length required for distance measurement as given in Equation (14).

## 4. Experimental Results

This section presents the training process of the improved lightweight object detection model, Ghost-YOLOv11n, followed by a comprehensive evaluation and analysis of its performance. Based on this detection model, experiments on UAV flight height estimation were conducted to validate the effectiveness and accuracy of the proposed monocular distance measurement method, which is grounded in the principle of similar triangles.

### 4.1. Ghost-YOLOv11n Model Training and Testing

#### 4.1.1. Training Details

Based on the dataset described in Section 3.1.1, the YOLOv11n.pt model was utilized as the pretrained weight to train and validate the following three models: (1) the original YOLOv11n model; (2) the Relu-YOLOv11n model, in which the original activation function was replaced with Relu() function; (3) the Ghost-YOLOv11n model, where the conventional convolution modules were replaced with Ghost modules. All training procedures were conducted in an Ubuntu 20.04 environment using a single NVIDIA GeForce RTX 3090 GPU with 24 GB of memory. The object detection networks were implemented and trained using the PyTorch (PyTorch 1.8.1) framework. The detailed training hyperparameters for the three models are summarized in Table 2.

#### 4.1.2. Evaluation Metrics

In object detection tasks, the Mean Average Precision (mAP) is one of the key metrics for evaluating model performance. Based on the prediction results, Precision (P) and Recall (R) can be calculated. P measures the accuracy of the model, defined as the proportion of correctly predicted target samples among all samples predicted as targets. R measures the completeness of the model, defined as the proportion of correctly detected targets among all true targets. The formulas for calculating P and R are given in Equations (15) and (16), respectively.

(17) $$P=\frac{TP}{TP+FP}$$

(18) $$R=\frac{TP}{TP+FN}$$

Here, TP + FP represents all positive samples detected by the model, including true positives (TP) and false positives (FP). A higher P indicates fewer false detections, meaning that most of the predicted targets are correct. Meanwhile, TP + FN denotes all ground truth targets in the dataset, including those correctly detected (TP) and those missed (FN). A higher R implies that the model can detect most targets, although it may also lead to an increase in false positives (FP).

To provide a balanced single metric that harmonizes both precision and recall, the F1-score (F1) is widely adopted. It is defined as the harmonic mean of P and R, offering a unified measure that is especially informative when seeking a trade-off between false positives and false negatives. The F_1_ is calculated as follows:

(19) $$F_{1}=\frac{2PR}{P+R}$$

Typically, there exists a trade-off between P and R, where increasing P may reduce R, and vice versa. Therefore, the precision–recall (PR) curve is plotted to comprehensively evaluate the detection performance of the model. For a single class, the average precision (AP) is defined as the area under the PR curve, calculated as follows:

(20) $$AP=\int_{0}^{1}P(R)dR$$

In practical computations, a discrete approximation method is commonly employed:

(21) $$AP=\frac{1}{m}\sum_{i=1}^{m}P(R_{i})$$

where R_i_ denotes different recall points, and P(R_i_) represents the corresponding precision values. The calculation of AP varies slightly across datasets. For instance, the PASCAL VOC dataset employs an 11-point interpolated average precision method, whereas the COCO dataset evaluates AP by averaging precision over all recall points. For multi-class object detection, the mAP is defined as the mean of AP values across all classes:

(22) $$mAP=\frac{1}{N}\sum_{i=1}^{N}AP_{i}$$

Here, N represents the total number of classes, and AP_i_ denotes the average precision of the i-th class. At different threshold levels, performance evaluation typically involves metrics such as mAP_0.5_ and mAP_0.5:0.95_ recall to assess the detection model’s effectiveness.

In practical object detection scenarios, besides model accuracy, the actual operational speed of the model is also a critical consideration. Frames Per Second (FPS) is a key metric used to evaluate the processing speed of a model, representing the number of image frames the model can process per second. The calculation formula for FPS is given by:

(23) $$FPS=\frac{N}{T}$$

where *N* represents the total number of processed frames, and *T* denotes the total time (in seconds) required to process these frames. A higher *FPS* indicates that the model can process input images more rapidly, thereby enhancing real-time detection capability.

#### 4.1.3. Model Performance Analysis

The performance metrics of the original YOLOv11n model, the Relu-YOLOv11n model (with ReLU activation function), and the Ghost-YOLOv11n model (with GhostConv module) are shown in Table 3. These three models differ in parameter scale and computational complexity. YOLOv11n serves as the baseline, Relu-YOLOv11n improves hardware deployment efficiency by optimizing the activation function, and Ghost-YOLOv11n reduces parameter count and computation cost while maintaining detection accuracy through a lightweight convolution structure. To comprehensively evaluate the performance of these models, experiments were conducted on three datasets: all classes, drone generalization, and self-built drone dataset. The results are shown in Table 3.

(1) All Classes Performance

As shown in Table 3(I), the baseline YOLOv11n model contains 2.58M parameters and 6.3 GFLOPs, achieving 97.7% P, 97.9% R, 98.7% mAP_0.5_, 97.80% F_1_ and 79.3% mAP_0.5:0.95_, with an inference speed of 8 FPS on the LuBancat4 board. The Relu-YOLOv11n model maintains the same parameter count and computation cost, while slightly improving P (97.9%), R(98.7%), F_1_(98.32%) and mAP_0.5_ (98.9%), with only a minor decrease in mAP_0.5:0.95_ (79.0%). Its inference speed increases to 12 FPS, reflecting a better trade-off between accuracy and speed. The Ghost-YOLOv11n model reduces parameters to 2.25 M and computation to 5.5 GFLOPs, achieving comparable accuracy (97.8% P, 98.4% recall, 98.12% F_1_, 98.8% mAP_0.5_, 79.5% mAP_0.5:0.95_) while significantly improving inference speed to 20 FPS, showing the best balance between lightweight efficiency and detection performance.

(2) Drone Generalization Performance

Table 3(II) presents the results on the drone generalization dataset. YOLOv11n achieves 95.7% P, 96.1% R, 97.9% mAP_0.5_, 95.88% F_1_ and 68.6% mAP_0.5:0.95_ at 8 FPS. Relu-YOLOv11n again improves the metrics, reaching 96.2% P, 97.6% R, 96.90% F_1_ and 98.3% mAP_0.5_, while maintaining the same mAP_0.5:0.95_ (68.6%), and increases inference speed to 12 FPS. Ghost-YOLOv11n achieves 96.0% P, 96.6% R, 96.31% F_1_, 98.1% mAP_0.5_, and 69.4% mAP_0.5:0.95_, with the highest FPS of 20. These results demonstrate that Ghost-YOLOv11n provides the most favorable balance of generalization accuracy and real-time performance, making it highly suitable for drone detection in open and complex environments.

(3) Drone Self-Built Dataset Performance

In Table 3(III), the self-built dataset results show all three models reaching extremely high accuracy. YOLOv11n achieves 99.8% P and R, 99.80% F_1_, with 99.4% mAP_0.5_ and 90.0% mAP_0.5:0.95_, at 8 FPS. Relu-YOLOv11n achieves nearly identical performance (99.7% P, 99.8% R, 99.75% F_1_, 99.4% mAP_0.5_, 89.5% mAP_0.5:0.95_), with speed improved to 12 FPS. Ghost-YOLOv11n maintains the same level of detection accuracy (99.6% P, 99.8% R, 99.70% F_1_, 99.4% mAP_0.5_, 89.7% mAP_0.5:0.95_), but significantly outperforms in inference speed at 20 FPS. This confirms that Ghost-YOLOv11n can achieve real-time detection without sacrificing accuracy, even under self-collected drone scenarios.

**Figure 9:**
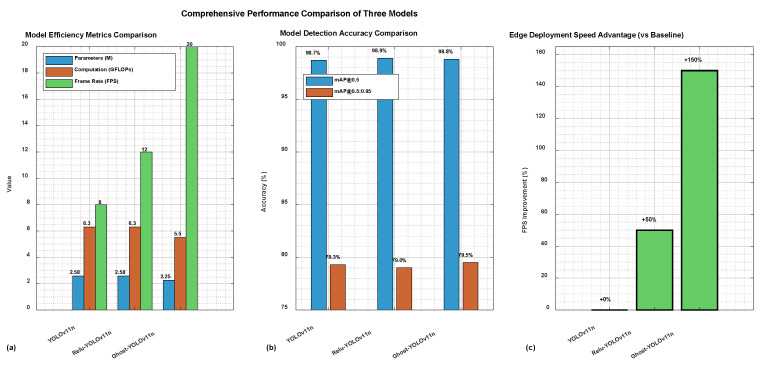
Comparative analysis of three models on the complete dataset. (**a**) Efficiency metrics (parameters, GFLOPs, and FPS). (**b**) Detection accuracy (mAP_0.5_ and mAP_0.5:0.95_). (**c**) FPS improvement relative to the baseline YOLOv11n.

To provide a more intuitive comparison of the models’ performance trade-offs, the data from Table 3(I) (complete dataset) is visualized in Figure 9. This multi-subplot figure includes: (a) a grouped bar chart comparing efficiency metrics (parameters, GFLOPs, and FPS), clearly showing Ghost-YOLOv11n’s superior speed (20 FPS) with reduced computational load; (b) an accuracy comparison chart for mAP_0.5_ and mAP_0.5:0.95_, confirming that all three models achieve high and comparable detection precision; and (c) a bar chart highlighting the FPS improvement percentage relative to the baseline, where Ghost-YOLOv11n achieves a 150% speed-up. This visualization reinforces that Ghost-YOLOv11n offers the optimal balance between lightweight design, detection accuracy, and real-time performance for edge deployment.

Overall, across all three evaluation scenarios (all classes, generalization, and self-built datasets), Ghost-YOLOv11n consistently achieves the best inference speed (20 FPS) with only a minimal reduction, if any, in accuracy compared to the original YOLOv11n and Relu-YOLOv11n models. The Relu-YOLOv11n offers a compromise with moderate accuracy and speed improvements. However, Ghost-YOLOv11n demonstrates the most favorable balance between lightweight design, detection accuracy, and real-time performance, making it the optimal choice for UAV detection tasks in resource-constrained edge environments.

**Figure 10:**
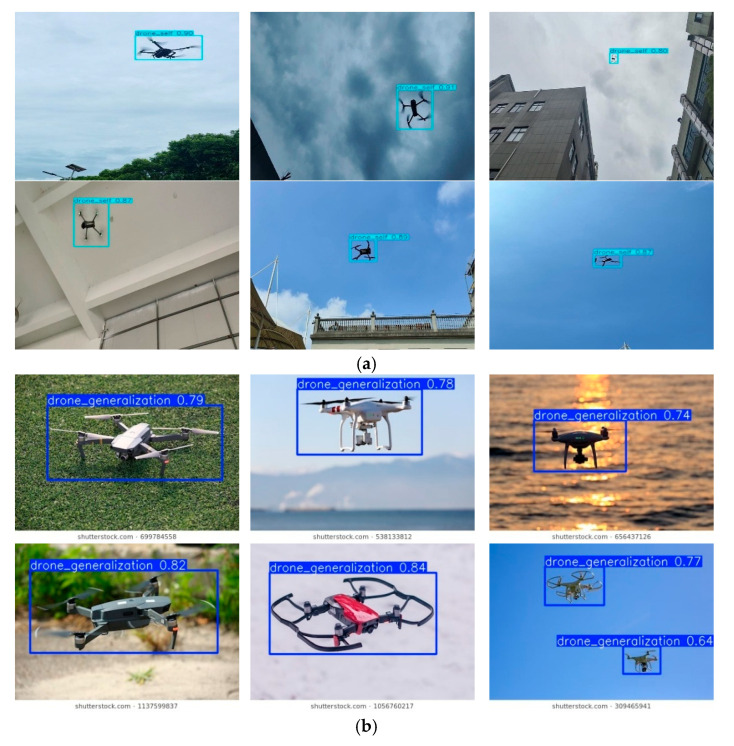
Detection Results of the Ghost-YOLOv11n Model. (**a**) Self-built subset. (**b**) Generalization subset [43].

As illustrated in Figure 10, the proposed Ghost-YOLOv11n model demonstrates robust detection performance for UAV targets under complex backgrounds, various viewing angles, and diverse lighting conditions.

To further demonstrate the advantages of the proposed model, we compared the training results of the Ghost-YOLOv11n model with several existing object detection models on the *VisDrone* dataset. The experimental results are presented in Table 4.

**Figure 11:**
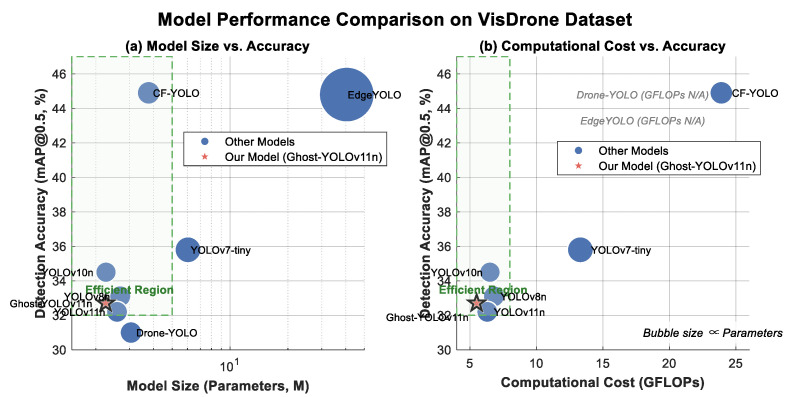
Accuracy–efficiency trade-off of various models on the VisDrone dataset. (**a**) Model Size vs. Accuracy. (**b**) Computational Cost vs. Accuracy.

Figure 11 illustrates the accuracy–efficiency trade-off among the evaluated models through two scatter plots, relating model size and computational cost (GFLOPs) to accuracy (mAP_0.5_), with bubble size representing parameter count. The plots highlight an “Efficient Region” balancing performance with resource constraints. Within this region, Ghost-YOLOv11n achieves an optimal balance, attaining competitive accuracy (32.7% mAP_0.5_, 19% mAP_0.5:0.95_) with minimal parameters (2.25 M) and the lowest computational cost (5.5 GFLOPs). This performance surpasses the baseline YOLOv11n and Drone-YOLO(Nano). In contrast, higher-accuracy models like CF-YOLO (44.9% mAP_0.5_) and EdgeYOLO (44.8% mAP_0.5_) reside outside this region due to their substantially greater computational (23.9 GFLOPs) or parametric (40.5 M) costs. While Ghost-YOLOv11n shows a minor accuracy gap compared to these models, it offers decisive advantages in efficiency. Compared to YOLOv8n (33.1% mAP_0.5_), it achieves comparable accuracy with significantly lower resource demands.

Overall, the evaluation demonstrates that Ghost-YOLOv11n excels in resource-limited settings by optimally balancing detection accuracy, model size, and computational load, making it particularly suitable for real-time UAV detection.

### 4.2. Drone Flight Height Measurement Experiment

**Figure 12:**
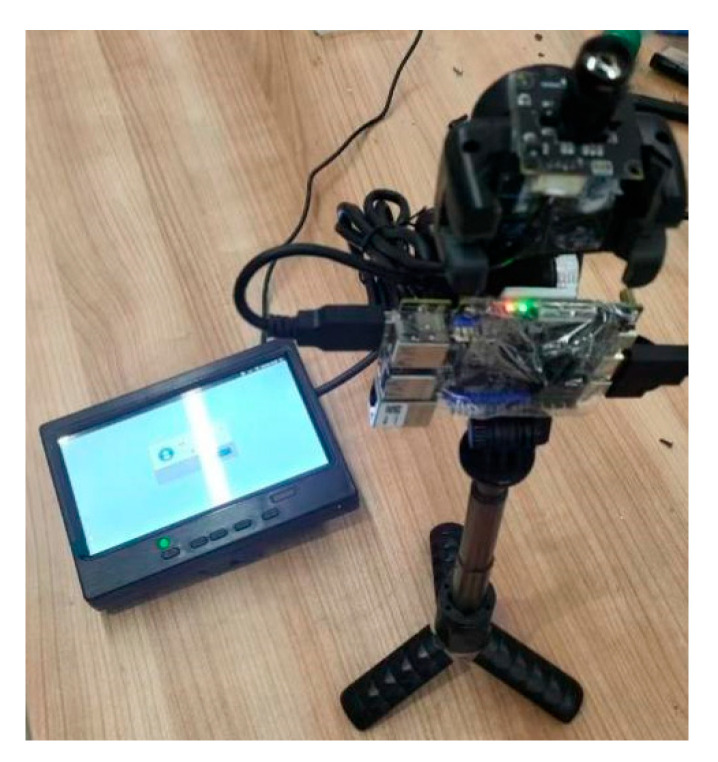
Experimental Apparatus.

To validate the accuracy of UAV height estimation, the Ghost-YOLOv11n model was converted to the .rknn format and deployed on the LuBancat4 development board. A Raspberry Pi USB camera (Zhongwei Aoke, Shenzhen, China) module with a 12 mm focal length lens was used to capture visual data during the experiments. The detection and distance information were displayed on a 10-inch YCXSQ-10 monitor (ZINCTUNG, Shenzhen, China) with a resolution of 1920 × 1080 pixels. To remove distortion in the image, the cv2.undistort() function in OpenCV was used for image correction. The distortion parameters were obtained from the camera calibration results in Section 3.3.3 and input into the function. The output image, after distortion removal, is more accurate for measurement purposes. As shown in Figure 12, the camera—facing upward—was mounted together with the LuBancat4 board on a tripod. The display was connected to the board via an HDMI cable to visualize the detection results and height measurements.

**Figure 13:**
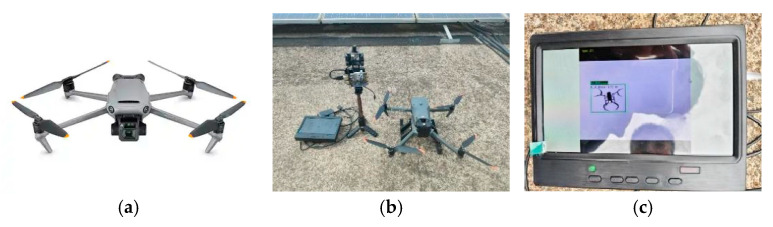
Height Measurement Experiment. (**a**) DJI 3E. (**b**) Experiment setup. (**c**) Distance Measurement Screen.

In the experiment, the DJI Mavic 3E (DJI, Shenzhen, China) was selected as the target UAV, with a wingspan width of 56 cm. The UAV was adjusted to hover at various height levels, and the estimated heights were compared with ground-truth measurements to evaluate the accuracy of the proposed method. The UAV used in the experiment and the corresponding test environment are shown in Figure 13.

To quantitatively evaluate the accuracy of the proposed system in measuring UAV flight height, the relative error is adopted as the evaluation metric. By comparing the estimated values with the actual height values, the measurement precision of the system can be assessed. The relative error *e_mea_* is defined as follows [46]:

(24) $$e_{mea}=\frac{\left|H_{real}-H_{mea}\right|}{H_{real}}\times 100\%$$

In the above equation, *H_real_* denotes the true height of the UAV above the ground, while *H_mea_* measured represents the estimated height obtained from the measurement.

To comprehensively validate the accuracy and robustness of the proposed algorithm, three sets of comparative experiments were designed to evaluate its performance under different environmental conditions. The first group was conducted under calm wind, few clouds, and moderate lighting to verify the height measurement accuracy in an ideal environment. The second group was carried out under windy, cloudy, and weak lighting conditions to assess the effects of wind disturbance and insufficient illumination on algorithm performance. The third group was performed under calm wind, few clouds, but strong lighting to examine the adaptability and stability of the algorithm in intense illumination environments. The results of the three groups are shown in Table 5, where Table 5(I)–(III) correspond to the first, second, and third groups, respectively. Table 5 presents the height measurement results under three different environmental conditions. In the first group with calm wind, few clouds, and moderate lighting, the algorithm achieved relatively high accuracy, with most relative errors within 10%, though larger deviations appeared at certain heights such as 10 m (23.1%), 15 m, 20 m, 50 m, and 60 m, indicating sensitivity to specific factors even under ideal conditions. In the second group with wind, cloudy skies, and weak lighting, the results showed greater fluctuations, especially at low heights (5–7 m, errors around 11%) and high heights (40 m at 25.83% and 50 m at 17.86%), while the performance at medium heights (9 m, 15 m, 20 m, and 30 m) remained relatively good with errors below 8% and as low as 0.65% at 20 m, suggesting that the algorithm maintains robustness in certain ranges but is more vulnerable at low and high heights under wind disturbance and insufficient light. It should be noted that under weak lighting with a 12 mm lens, the algorithm could stably recognize UAV targets up to around 50 m, while recognition beyond this distance became significantly more difficult. Although UAVs above 50 m could still occasionally be detected (e.g., at 60 m), the recognition rate and stability dropped markedly. In the third group with calm wind, few clouds, and strong lighting, the algorithm performed more stably, with most errors below 10% and particularly low values at 6 m (0.33%) and 15 m (1.73%), but higher errors appeared at 7 m, 8 m, and 20 m (7.43%, 8.50%, and 8.25%), likely due to reflections or contrast variations under strong illumination. Notably, when the height exceeded 30 m, the algorithm failed to detect the UAV, presumably because strong sunlight reduced the contrast between the UAV and the sky background, resulting in recognition failure. Overall, the main sources of error can be summarized as follows: under windy conditions, UAV motion causes instability of the detection bounding box, leading to fluctuations in size and thus distance estimation errors; at higher heights, rotor blades and structural details are difficult to distinguish, reducing detection accuracy and causing measurement deviations; and under strong lighting, the reduced contrast between the UAV and the background prevents stable recognition above 30 m. In conclusion, the effective recognition height limit was approximately 50 m under weak lighting (with a 12 mm lens) and around 30 m under strong lighting. In summary, the proposed method demonstrates reasonable robustness across different environments.

### 4.3. Power Consumption Test Experiment of LubanCat-4 Development Board

**Figure 14:**
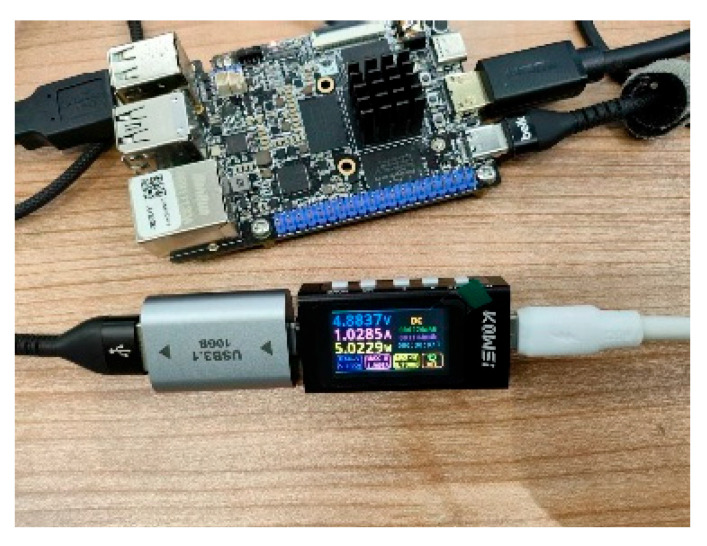
Power Consumption Test Experiment.

To evaluate the power consumption of the proposed system and its practical benefits, we conducted comparative experiments on the LuBanCat4 platform under different operating conditions, as illustrated in Figure 14. Power measurements were obtained using a KWS-X1 Type-C USB power meter. The results, summarized in Table 6, demonstrate the energy efficiency of Ghost-YOLOv11n compared to other lightweight models when tested on the VisDrone dataset. Our model consumes 4.2 W at 10 FPS, yielding an energy cost of 0.42 J per frame. In contrast, YOLOv8n (4.7 W, 10 FPS) and YOLOv11n (4.6 W, 8 FPS) require 0.47 J and 0.575 J per frame, respectively. This superior energy efficiency translates directly into extended operational endurance: using a typical 20,000 mAh portable battery (≈100 Wh), Ghost-YOLOv11n can sustain continuous active detection for approximately 24 h, compared to about 21 h for YOLOv8n and 17 h for YOLOv11n. Although Ghost-YOLOv11n exhibits a modest decrease in mAP relative to some larger models, its significantly lower energy per frame and longer battery life affirm that the trade-off in accuracy yields meaningful practical advantages for resource-constrained, long-duration ground-based surveillance.

## 5. Discussion

### 5.1. Comparative Analysis of Distance Measurement Methods

This study proposes a monocular vision-based UAV target distance measurement method using the principle of similar triangles, which differs significantly from the approaches in [19,20] in both target type and detection strategy. It is noteworthy that the core ranging principle and EKF-based tracking framework are built upon our previous work on ADG-YOLO [3], but are specifically adapted here for the ground-to-air monitoring scenario. In contrast, the proposed method offers two key advantages:

(1) Adaptability to Target Types and Modeling Approaches:

The methods in [19,20] primarily focus on distance estimation for vehicle targets, where the models are typically constructed based on the rear projection area, vanishing lines, or the pixel height of the object. However, these approaches encounter limitations when applied to aerial targets such as UAVs. Due to the highly variable projection shapes and sizes of UAVs in images, the heuristic models developed for vehicles are difficult to generalize. For example, the rear projection area used in [19] is not applicable to UAVs with more complex 3D structures. The vanishing line method in [20] relies on ground-plane constraints, which are not well-defined for aerial targets, particularly since UAVs operate at varying altitudes.

(2) Differences in Target Detection Methodology:

Traditional methods, such as those in [19], assume the availability of specific pixel-level information about the target, such as its bottom position or bounding box boundaries. These assumptions greatly reduce adaptability in dynamic scenes where target position and appearance vary frequently. While [19] employs the Mask R-CNN instance segmentation network to obtain precise pixel-level target regions, the approach suffers from slow inference speed and high computational cost, making it unsuitable for real-time distance estimation.

The novelty of this work lies in integrating YOLOv11 with an Extended Kalman Filter (EKF) for geometric modeling. YOLOv11 is used to detect UAV targets in real time, and EKF is applied to model the target dynamics and perform distance estimation. The proposed method achieves high computational efficiency, strong adaptability, and robust accuracy, making it particularly well-suited for real-time distance estimation of dynamically flying UAVs.

### 5.2. Discussion on the Comparison of YOLO Models

In this study, Ghost-YOLOv11n demonstrates an excellent balance between computational efficiency and accuracy, making it particularly suitable for applications where real-time performance and computational resources are crucial. This model represents a distinct architectural evolution from our prior ADG-YOLO [3], focusing on a unified GhostConv design for enhanced deployment stability on fixed ground nodes, rather than the combined C3Ghost plus ADown modules used in the airborne-oriented [3]. Compared to other YOLO models, Ghost-YOLOv11n offers the following significant advantages:

(1) Lower computational resource consumption:

Ghost-YOLOv11n is more efficient in terms of parameter count and computational cost compared to other models, such as YOLOv7-tiny, YOLOv8n and YOLOv10n. Ablation against the baseline YOLOv11n shows that the GhostConv module reduces parameters and GFLOPs by 12.7% while increasing edge inference speed by 150% (from 8 to 20 FPS), with no loss in mAP_0.5_ (98.8%). Specifically, Ghost-YOLOv11n has 2.25 M parameters and 5.5 GFLOP computational cost, while YOLOv10n has 2.26 M parameters and 6.5 GFLOP. Although YOLOv7-tiny and YOLOv8n have lower computational costs (6.04 GFLOP and 6.8 GFLOP, respectively), Ghost-YOLOv11n still consumes fewer resources than these larger models [37]. Importantly, the performance of Ghost-YOLOv11n has not been significantly compromised, and it delivers superior computational efficiency in practical applications.

(2) Stable accuracy performance:

In terms of accuracy, Ghost-YOLOv11n achieves mAP_0.5_ and mAP_0.5:0.95_ scores of 32.7% and 19%, respectively, which are comparable to YOLOv7-tiny (35.8% and 18.8%) and YOLOv8n (33.1% and 19.2%) [37]. This indicates that Ghost-YOLOv11n maintains accuracy with minimal reduction in performance, even with lower computational costs. This highlights the well-balanced trade-off between accuracy and computational resource consumption. Notably, compared to YOLOv11n (32.2% and 18.6%), Ghost-YOLOv11n offers a slight improvement in accuracy while reducing computational costs.

(3) Optimized resource utilization:

Ghost-YOLOv11n optimizes both computational and memory usage by adopting the lightweight GhostNet architecture. It shows a significant advantage over YOLOv7-tiny (6.04 M parameters, 13.3 GFLOP) in terms of parameter count and computational cost, with minimal accuracy loss [37]. Specifically, while YOLOv7-tiny achieves an *mAP*_0.5:0.95_ of 18.8%, Ghost-YOLOv11n provides a comparable accuracy (*mAP*_0.5:0.95_ of 19%) with a relatively lower computational cost (5.5 GFLOP), making it more efficient in resource-constrained environments.

(4) Comparison with larger models:

Compared to high-performance models like EdgeYOLO (40.5 M parameters) and CF-YOLO (3.77 M parameters, 23.9 GFLOP), Ghost-YOLOv11n still offers a notable advantage in computational efficiency. Although EdgeYOLO and CF-YOLO achieve higher accuracy in mAP_0.5_ and mAP_0.5:0.95_, with scores of 44.8% and 26.4%, and 44.9% and 27.5%, due to their high computational overhead, these two models exhibit limited inference speed on resource-constrained devices, making it difficult to meet real-time performance requirements [37,40]. While Ghost-YOLOv11n has slightly lower accuracy, its lower computational requirements make it a more feasible solution for resource-limited environments, particularly in UAV target detection applications, where it provides highly satisfactory performance.

In conclusion, Ghost-YOLOv11n significantly reduces computational burden while maintaining high accuracy, making it particularly suitable for deployment on embedded systems or edge devices. Its balance between accuracy and computational efficiency makes it a competitive choice, especially for real-time inference tasks in UAV target detection applications.

### 5.3. Error Analysis for UAV Altitude Measurement

The system demonstrates high altitude measurement accuracy (error < 10%) within 30 m. However, the error increases to 16–26% at distances of 40–60 m. Based on experimental observations and geometric model analysis, we identify two primary factors contributing to the increased error at long range:

Bounding-Box Quantization and Inflation Effect at Low Resolution: The accuracy of the similarity-triangle-based ranging model critically depends on the precise measurement of the target’s projected width *w*. When the UAV is at a significant distance, its pixel size in the image diminishes sharply (e.g., occupying only 15–20 pixels in width at 50 m). At this scale, the inherent anchor mechanism and the Non-Maximum Suppression (NMS) process of the YOLO detector lead to discretization and a systematic slight inflation of the bounding box size. Specifically, to ensure the recall rate for small objects, the network tends to output bounding boxes that are slightly larger than the actual target. This limited “pixel-level quantization” bias has a negligible impact at close range (where the target’s pixel size is large). However, at long distances, because *w* is in the denominator of the geometric formula, minor pixel errors are significantly amplified by the model, resulting in a larger percentage error in the calculated altitude *H*.

Feature Degradation and Identification Bias for High-Altitude Targets: At low or medium altitudes, the clear structure of the UAV body (such as propellers and fuselage) provides rich features for the detection network. As altitude increases, the resolution of the target in the image decreases, causing fine, discriminative structures (especially the fast-rotating propellers) to gradually blur and become difficult for the network to capture effectively. Consequently, the detector relies more on the overall silhouette or the most prominent parts of the target for identification. Therefore, the bounding box predicted under high-altitude conditions may correspond more closely to the core body of the UAV rather than its full width including the propellers. This directly leads to a projected width *w* used in calculation that is smaller than the actual physical width *W*, thereby systematically overestimating the true flight altitude of the UAV.

Consideration of Other Potential Error Sources: Our study also considered other potential factors that may influence ranging accuracy, primarily camera calibration errors and target pose-induced distortion. Camera calibration errors were effectively minimized through a rigorous offline calibration procedure, yielding a mean reprojection error of approximately 0.1 pixels. Sensitivity analysis indicated that within the effective ranging distance of 30 m, their residual influence was relatively minor compared to errors introduced by detector performance. However, at greater distances, the geometric amplification effect of such errors becomes increasingly significant. Drone pose-induced projection width distortion represents an inherent limitation of monocular vision-based geometric ranging methods. This experiment primarily focused on hovering or steadily flying targets with relatively small variations in yaw and pitch angles; thus, this factor was not a major source of error in the present study. Nevertheless, in complex real-world flight scenarios, severe pose variations could lead to significant ranging deviations, which constitutes a key challenge to be addressed in future research.

In summary, the long-range measurement error stems from two combined factors: pixel-level bounding-box inflation and high-altitude feature degradation. The latter dominates under high-altitude conditions, leading to systematically overestimated measurements beyond 40 m. Together with other minor factors, these represent inherent challenges of monocular geometric ranging. Future work may explore super-resolution reconstruction, small-object-optimized detection heads, or pose-aware projection models to mitigate these limitations.

### 5.4. Limitations and Future Directions

Although the distance measurement method proposed in this paper demonstrates promising performance in specific application scenarios, it still has certain limitations. Firstly, the current approach relies on known drone target sizes, which restricts its applicability across diverse scenarios. This limitation is consistent with our earlier ADG-YOLO system [3], highlighting a common challenge in geometry-based monocular ranging that requires prior knowledge of target dimensions. Specifically, this study only measures the flight altitude of drones without considering size variations among different drone types. Consequently, the method may encounter adaptability issues when dealing with drones of varying sizes and shapes, particularly in real-world applications where drone target dimensions exhibit significant variability.

Secondly, the current deployment validation is conducted primarily on the LuBanCat4 platform equipped with a dedicated NPU. While the Ghost-YOLOv11n model is designed with lightweight principles (e.g., reduced parameters and GFLOPs), its suitability for deployment on devices with significantly poorer computational capabilities (e.g., those lacking an NPU or with very limited CPU power) requires further investigation. On such devices, achieving real-time performance might necessitate additional adaptations, such as more aggressive model quantization, pruning, or reduction of input image resolution, which could impact detection range or accuracy. This defines the practical deployment boundary of the current work.

Thirdly, the influence of training data variation on the obtained results was not systematically investigated. While our hybrid dataset (combining self-collected and public data) provided diversity in UAV types and backgrounds, we did not perform controlled ablation studies to quantify how changes in training data scale, composition, or class distribution would affect model performance. This is an acknowledged limitation, as the model’s generalization capability to significantly different data domains remains partially unverified.

Additionally, the study lacks comparative experiments with other lightweight models on the LuBanCat4 development board, such as recent small models including Drone-YOLO (Nano) [36] and LMWP-YOLO [37]. This omission limits the evaluation of the proposed model’s deployment efficiency relative to emerging alternatives in resource-constrained hardware environments.

Future research directions include building a comprehensive dataset that incorporates UAVs of different types and sizes to expand the applicability of the distance measurement method. Such a dataset would enhance the robustness of the model under diverse targets, ensuring adaptability to a variety of flight scenarios. Furthermore, we will systematically investigate how variations in training data composition, scale, and distribution affect model performance through controlled ablation studies. Moreover, future work should also focus on further lightweighting the model to achieve more efficient real-time distance measurement in resource-limited environments. At the same time, with dataset expansion and model optimization, improving measurement accuracy—particularly under dynamic flight conditions—will be a key objective. Additionally, future research will incorporate tests under angle variations and noise interference to validate and enhance the algorithm’s robustness in realistic environments. To address the above limitations, we also plan to reproduce these comparative lightweight models and establish a deployment pipeline on the LubanCat4 development board, enabling comprehensive performance benchmarking.

## 6. Conclusions

This study presents a ground-based visual system for real-time UAV detection and altitude measurement, thereby completing a dual-node perception suite alongside our earlier airborne-oriented ADG-YOLO framework [3]. While leveraging the proven EKF tracking and similar-triangle ranging principles from [3], this work is fundamentally re-optimized for the static ground station scenario, prioritizing long-term stability and power efficiency over raw performance. The cornerstone is Ghost-YOLOv11n, a detector that employs a unified GhostConv design to reduce computational cost by 12.7% while achieving 98.8% mAP_0.5_. When deployed on an LuBanCat4 edge device, the system delivers 20 FPS and reliable height estimation with errors typically below 10% within 30 m, albeit with reduced accuracy at longer ranges (16–26% error at 40–60 m). With a power draw of only 5 W, it enables sustained field operation, validating its practicality for guarding fixed infrastructure.

Limitations and Future Work: A key limitation, inherited from the geometric approach in [3], is the dependency on known UAV dimensions. Future work will pursue model-agnostic ranging, dataset expansion, and further model lightweighting. We also plan to benchmark against contemporaries like Drone-YOLO [38] on various edge hardware. By advancing this ground-focused branch, we aim to build a holistic UAV perception ecosystem that seamlessly integrates both mobile (airborne) and fixed (ground) intelligent nodes, as initiated by [3] and this work.

## Figures and Tables

**Table 1:** Comparison of Edge Computing Devices for Drone Detection Applications.

Device Name	Computing Power	Power Consumption	Price (USD)
Lubancat 4	6 TOPS	~5 W	100~150
NVIDIA Jetson Nano	0.5 TOPS	5–10 W	200~300
NVIDIA Jetson TX2	1.3 TOPS	7.5–15 W	290~420
NVIDIA Jetson Xavier NX	21 TOPS	10–15 W	430~800

**Table 2:** YOLOv11 Model Training Hyperparameter Settings.

Parameter Settings	Value Selection
Activation Function	Silu()/Relu()
batch size	16
Initial Learning Rate	1 × 10^−5^
epochs	500

**Table 3:** (**I**). Model Performance (all classes). (**II**). Model Performance (drone_generalization). (**III**). Model Performance (drone_self).

Model	Para (M)	GFLOPs (G)	P (%)	R (%)	F_1_ (%)	mAP_50_ (%)	mAP_50:95_ (%)	FPS
**(I)**								
YOLOv11n	2.58	6.3	97.7	97.9	97.80	98.7%	79.3%	8
Relu-YOLOv11n	2.58	6.3	97.9	98.7	98.32	98.9%	79.0%	12
Ghost-YOLOv11n	2.25	5.5	97.8	98.4	98.12	98.8%	79.5%	20
**(II)**								
YOLOv11n	2.58	6.3	95.7	96.1	95.88	97.9%	68.6%	8
Relu-YOLOv11n	2.58	6.3	96.2	97.6	96.90	98.3%	68.6%	12
Ghost-YOLOv11n	2.25	5.5	96.0	96.6	96.31	98.1%	69.4%	20
**(III)**								
YOLOv11n	2.58	6.3	99.8%	99.8%	99.80	99.4%	90.0%	8
Relu-YOLOv11n	2.58	6.3	99.7%	99.8%	99.75	99.4%	89.5%	12
Ghost-YOLOv11n	2.25	5.5	99.6%	99.8%	99.70	99.4%	89.7%	20

**Table 4:** Comparison experiments of different models on the VisDrone dataset.

Model	mAP_50_	mAP_50:95_	Para (M)	GFLOP (G)
YOLOv7-tiny [37]	35.8%	18.8%	6.04	13.3
YOLOv8n [37]	33.1%	19.2%	2.68	6.8
YOLOv10n [37]	34.5%	19.9%	2.26	6.5
YOLOv11n [37]	32.2%	18.6%	2.58	6.3
Drone-YOLO(Nano) [38]	31.0%	17.5%	3.05	-
EdgeYOLO [40]	44.8%	26.4%	40.5	-
CF-YOLO [37]	44.9%	27.5%	3.77	23.9
Ghost-YOLOv11n (ours)	32.7%	19.0%	2.25	5.5

**Table 5:** (**I**). Height measurement results (Calm, Clear, Moderate Light). (**II**). Height measurement results (Windy, Cloudy, Low Light). (**III**). Height measurement results (Calm, Clear, Strong Light).

Experiment ID	H_real_ (m)	H_mea_ (m)	e_mea_
**(I)**			
1	5	5.26	5.20%
2	6	6.19	3.17%
3	7	6.85	2.14%
4	8	8.19	2.37%
5	10	12.31	23.10%
6	15	16.72	11.47%
7	20	23.83	19.15%
8	30	27.36	8.80%
9	40	37.33	6.68%
10	50	58.69	17.38%
11	60	70.00	16.67%
**(II)**			
1	5	5.60	12.00%
2	6	6.71	11.83%
3	7	7.78	11.14%
4	9	9.11	1.22%
5	10	10.77	7.70%
6	15	14.18	5.47%
7	20	20.13	0.65%
8	30	30.27	0.90%
9	40	50.33	25.83%
10	50	58.93	17.86%
**(III)**			
1	5	5.25	5.00%
2	6	6.02	0.33%
3	7	7.52	7.43%
4	8	8.68	8.50%
5	9	9.57	6.33%
6	10	10.2	2.00%
7	15	15.26	1.73%
8	20	18.35	8.25%
9	30	32	6.67%

**Table 6:** Comparison of Lightweight Detection Models in power (Visdrone dataset).

Model	FPS	Power Consumption (W)	Energy Per Frame (J)
YOLOv8n	10	4.7	0.470
YOLOv11n	8	4.6	0.575
Ghost-YOLOv11n (ours)	10	4.2	0.420

## Data Availability

The original datasets presented in this study are available on request from the corresponding author.
